# Supplementary material for: Chitosan-Based Structural Color Films for Humidity Sensing with Antiviral Effect
Source: Nanomaterials (Basel). 2024 Feb 13;14(4):351. doi: 10.3390/nano14040351 (PMC10892554; doi:10.3390/nano14040351)
Supplement: Supplementary file 1 [file nanomaterials-14-00351-s001.zip › nanomaterials-2825582-supplementary.pdf]

Supporting Information

Chitosan-Based Structural Color Films for Humidity Sensing with Antiviral Effect

Darya Burak[1, 2, ‡], Dong-Chan Seo[3,4 ‡], Hong-Eun An[1,5], Sohee Jeong[1], Seung Eun Lee[3,*], and So-Hye Cho[1, 2, *]

[1] Materials Architecturing Research Center, Korea Institute of Science & Technology, 5 Hwarang-ro 14-gil, Seongbuk-gu, Seoul 02792, Republic of Korea

[2] Department of Nanomaterial Science and Engineering, Korea University of Science and Technology, 217 Gajeong-ro Yuseong-gu, Daejeon 34113, Republic of Korea

[3] Research Animal Resources Center, Korea Institute of Science & Technology, 5 Hwarang-ro 14-gil, Seongbuk-gu, Seoul 02792, Republic of Korea

[4] School of Biosystem and Biomedical Science, College of Health Science, Korea University, 145 Anam-ro, Seongbuk-gu, Seoul 02841, Republic of Korea

[4] Department of Materials Science and Engineering, College of Engineering, Korea University, 145 Anam-ro, Seongbuk-gu, Seoul 02841, Republic of Korea

[‡] Contributed equally

* Correspondence: S-H.Cho: sohyec@kist.re.kr; S.E.Lee: selee@kist.re.kr

Methods

The antiviral assays procedure was adapted from our previous study [1] with slight modifications. Cell culture and lentiviral production procedures were identical to that described in [1]. Modified conditions and parameters of the antiviral assays are highlighted *in italics.*

Antiviral Testing of chitosan and chitosan/Ag Films. The antiviral effect of chitosan and chitosan/Ag films was investigated on the cell cultures infected with lentivirus (*pCDH*-CMV-mCherry). Lentivirus belongs to a class of retroviruses that have an RNA genome. Since SARS-CoV-2 virus has limitations as a hostile infectious disease, its use is strictly restricted to biosafety level 3. On the other hand, lentivirus can be safely mass-produced at biosafety level 2, making it possible to utilize it for laboratory-scale screening. Lentivirus also has a high infection rate (>90 %), high protein expression levels, and can be employed both *in vitro* and *in vivo*.

The antiviral effect of chitosan and chitosan/Ag was examined by *two* different methods: virus infectivity assay (fluorescent protein expression (mCherry) of virus-infected cells), real-time PCR assay.

(1) Virus infectivity assay

Two volumes, 1 μL each, of *pCDH*-CMV-mCherry (1 x 10^7 Genome Copy (GC)/mL) were drop-cast on the films and left to dry (react) for *20 min*. Then, the residual virus was collected from the films by pipetting each viral point with *4* μL of PBS (phosphate-buffered saline) twice (total of *8* μL). From there, the collected virus was transferred into two sterilized 6-well plates (two viral points) which were seeded with HeLa cells (0.7 x 10^5 cells/well) and incubated for 72 h at 37 °C in a 5% CO_2 incubator. Duplication of this assay by placing two volumes of the virus per sample surface was performed to ensure the reliability of the results. The viral infection was analyzed by fluorescence microscopy (CKX53, Olympus). Fluorescent cell counting of the images was then performed in ImageJ Software (ImageJ, NIH). The tests were repeated for four times (n = 4) in this study.

(2) Real-time PCR assay

1 μL of *pCDH*-CMV-mCherry (1 x 10^7 GC/mL) was drop-cast on the films and left to dry (react) for *20 min*. Then, the residual virus was collected from the films by pipetting with *2.5* μL of PBS solution twice (total of *5* μL). The virus-suspended solutions were sampled with DNase I buffer, DNase I, and DI. From there, the viral genome was amplified to titrate viral DNA by real-time PCR (*A28567*, Applied Biosystems). The tests were repeated four times (n = 4) in this study.

Statistical Analyses. Statistical analyses were performed using Prism 9 (GraphPad Software, Prism 9, Cal-ifornia, USA). For the comparison of multiple groups, nonparametric repeated-measure one-way analysis of variance (ANOVA) with Dunn's multiple comparison test compared with control samples (silicon wafer). $p < 0.05$ was considered to indicate statistical significance throughout the study. The significance level is represented as asterisks (* $p < 0.05$; ns, not significant). All data are presented as mean ± SEM.

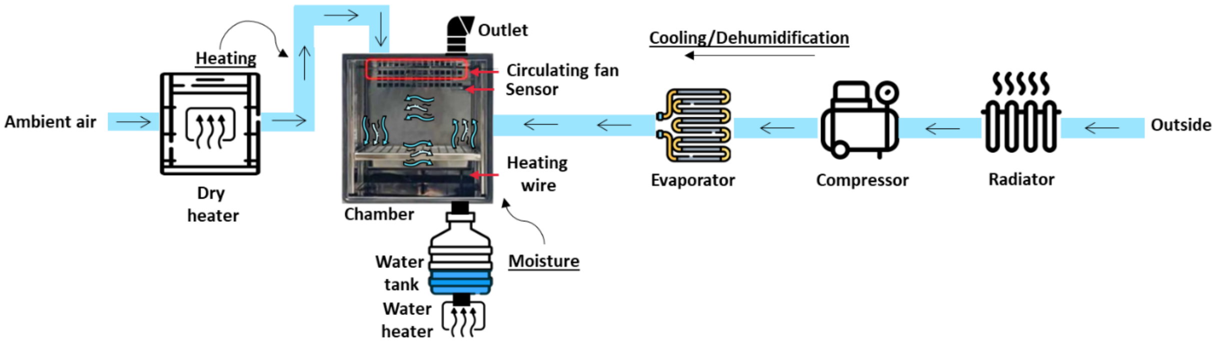

Figure S1. Schematic diagram of the humidity generation system.

<u>Humidity chamber working principle:</u> The humidifier heater in the water tank generates heat, causing the water to boil and release moisture particles (water vapor) into the chamber. Adjustments in water temperature and condensation from the refrigerant contribute to controlling and maintaining optimal humidity conditions. Furthermore, dehumidification is achieved through a cooling and ventilation system. This system, powered by a compressor-evaporator, cools the chamber, causing the principle of condensation to come into effect and effectively reducing humidity levels. Simultaneously, natural ventilation aids in the gradual removal of humidity. The chamber's ventilation system is a crucial component in this process, automatically regulating the amount of airflow. This responsive mechanism ensures efficiency in adapting to changes in humidity levels, creating a controlled and stable experimental environment.

<u>Calibration method:</u> The calibration of the equipment (temperature and humidity control chamber, S-TH31, SERIMA) was performed by the manufacturer. The manufacturer's stated accuracy for humidity is within +- 2%, and the equipment's calibration was verified using a calibrated humidity reference standard during installation in our laboratory. This comparison demonstrated negligible differences between the sensor readings and the reference measurements.

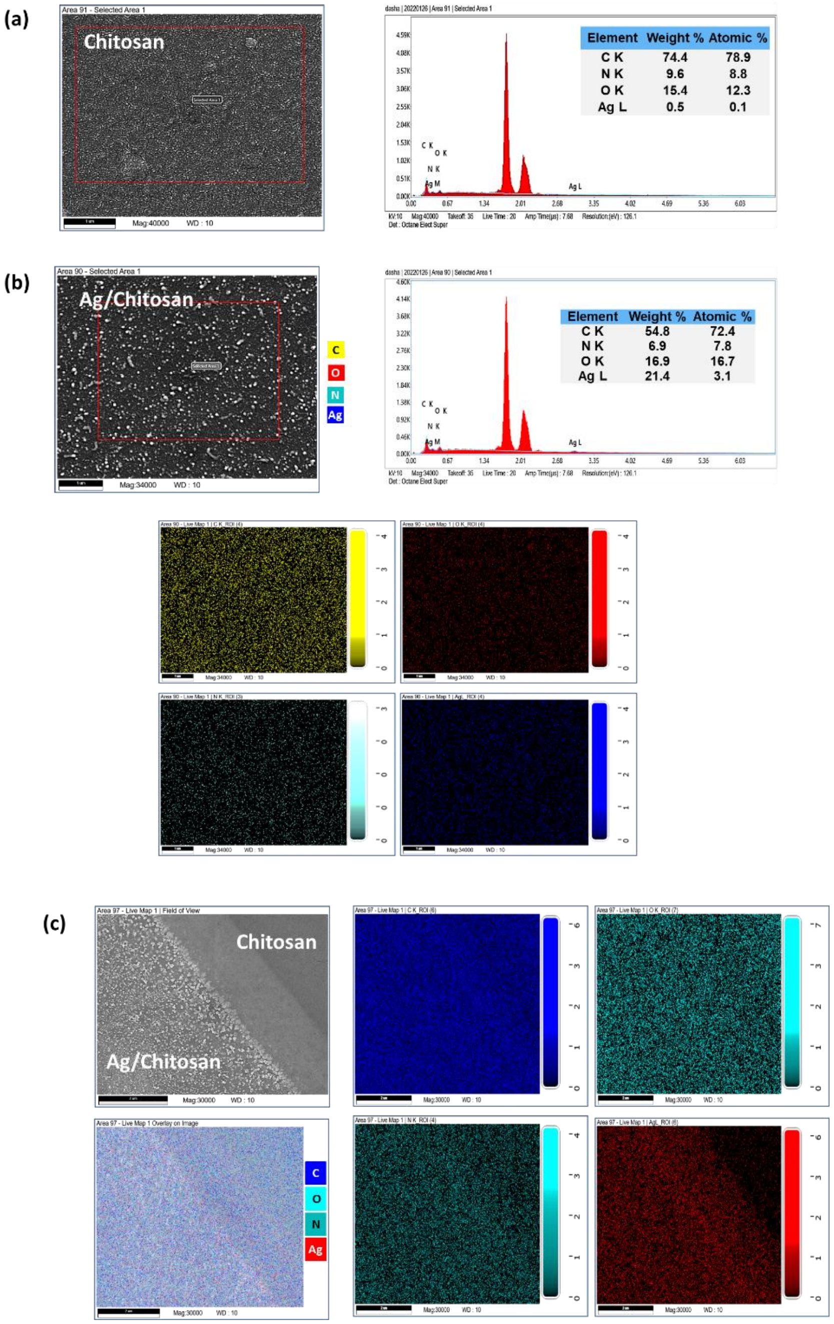

Figure S2. EDS analysis of chitosan (a) and EDS and mapping analysis of Ag/chitosan (b) films. To demonstrate a greater contrast between pure chitosan and silver-deposited chitosan surface, SEM images were taken at a boundary of two surfaces for (c).

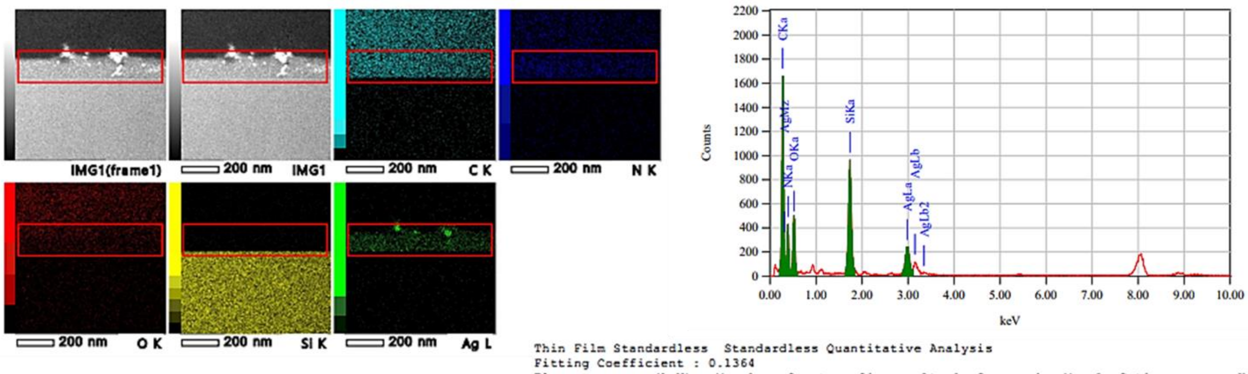

Thin Film Standardless Standardless Quantitative Analysis
Fitting Coefficient : 0.1364

Element	(keV)	Mass%	Counts	Sigma	Atom%	Compound	Mass%	Cation	K
C K (Ref.)	0.277	45.82	7414.82	0.63	66.63				1.0000
N K !	0.392	7.34	1756.92	0.28	9.15				0.6760
O K !	0.525	7.90	2500.38	0.29	8.62				0.5110
Si K !	1.739	20.22	6826.60	0.56	12.57				0.4793
Ag L	2.984	18.72	2947.41	0.61	3.03				1.0277
Total		100.00			100.00				

Figure S3. TEM EDS and mapping of the cross-section of Ag/chitosan film.

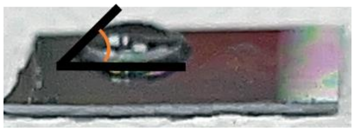

Chitosan WCA 55°

Figure S4. Water contact angle (WCA) of chitosan. WCA was measured at 55°, indicating the hydrophilicity of the chitosan film.

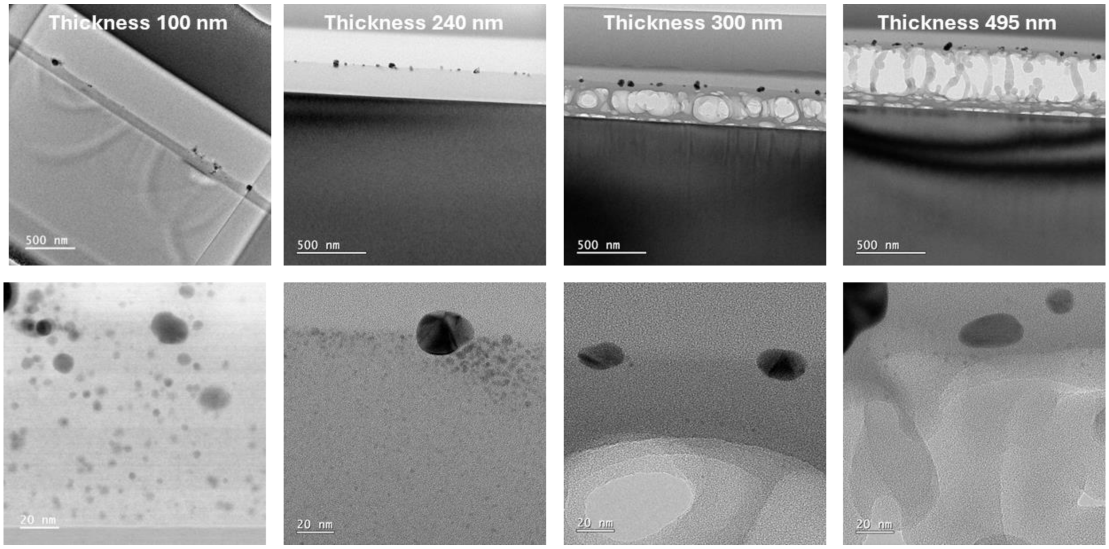

Figure S5. TEM images of a cross-section of the Ag/chitosan films with chitosan thickness of 100, 240, 300, and 495 nm. Bottom high-resolution images correspond to above images with the detailed thicknesses. Chitosan films of 300 and 495 nm underwent visible damage due to beam exposure during TEM measurements.

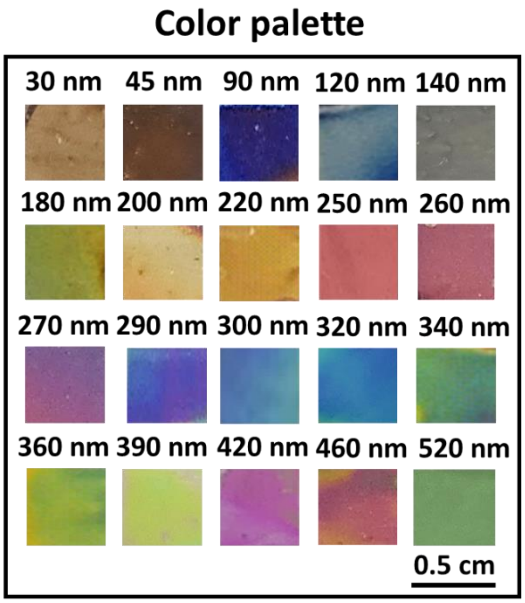

Figure S6. Color palette of the fabricated chitosan films (images taken via camera).

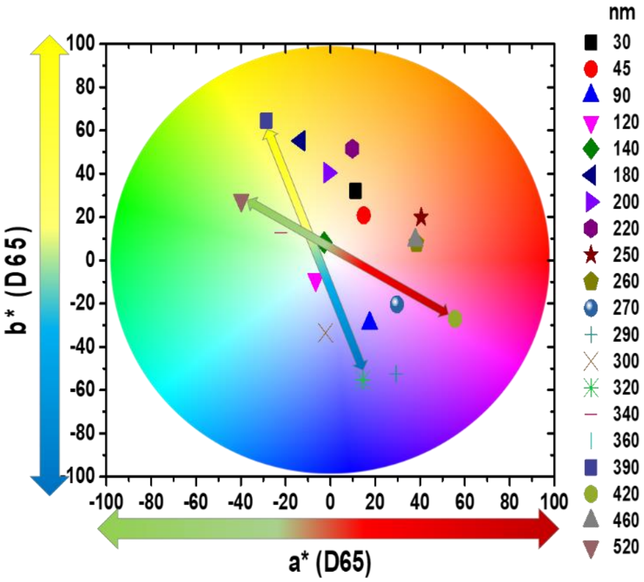

Figure S7. CIELAB (a*b*) coordinate diagram, where a* and b* parameters were derived from the color characterization of the chitosan films presented in Figure S5. Arrows indicate colors with highest saturation: warm yellow and dark blue (a*); pink magenta and green (b*).

Table S1. CIELAB (L*a*b*) color parameters of a chitosan film exposed to different RH (relative humidity) levels.

Chitosan film	Color parameters			
Humidity	L*	a*	b*	Color
25%	71.32	19.13	0.01	
45%	68.99	12.59	-20.21	
65%	70.92	5.59	-19.90	
75%	78.95	-15.03	-2.19	
90%	81.07	-14.60	12.09	

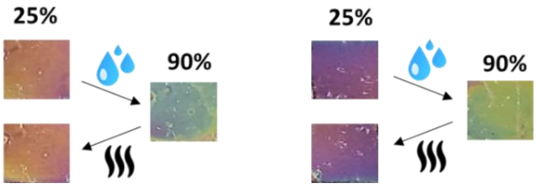

Figure S8. Reversible color change of chitosan film from 25% to 90% RH, and vice versa.
